# “He Fell in and That's How He Became a Fossil!”: Engagement With a Storytelling Exhibit Predicts Families' Explanatory Science Talk During a Museum Visit

**DOI:** 10.3389/fpsyg.2021.689649

**Published:** 2021-08-19

**Authors:** Maureen A. Callanan, Claudia L. Castañeda, Graciela Solis, Megan R. Luce, Mathew Diep, Sam R. McHugh, Jennifer L. Martin, Judy Scotchmoor, Sara DeAngelis

**Affiliations:** ^1^Department of Psychology, University of California, Santa Cruz, Santa Cruz, CA, United States; ^2^Department of Psychology, Loyola University Chicago, Chicago, IL, United States; ^3^Children's Discovery Museum of San Jose, San Jose, CA, United States; ^4^Education and Public Outreach, University of California Museum of Paleontology, University of California, Berkeley, Berkeley, CA, United States; ^5^Exhibition and Experience Design Program, Fashion Institute of Technology, New York, NY, United States

**Keywords:** storytelling, science, informal science learning, parent-child conversation, museum learning, children's science understanding

## Abstract

Parent-child conversations in everyday interactions may set the stage for children's interest and understanding about science. Studies of family conversations in museums have found links to children's engagement and learning. Stories and narratives about science may spark children's interest in science topics. This study asks whether a museum exhibit that provides opportunities for families to create narratives might encourage families' explanatory science talk throughout the rest of the exhibit. The project focused on the potential impact of a hands-on story-telling exhibit, the “spin browser” embedded within a larger exhibition focused on fossilized mammoth bones—*Mammoth Discovery!* at Children's Discovery Museum of San Jose. Participants were 83 families with children between 3 and 11 years (mean age 7 years). We coded families' narrative talk (telling stories about the living mammoth or the fossil discovery) and connecting talk (linking the story to other nearby exhibits) while families visited the spin browser, and we also coded families' explanatory science talk at the exhibits that contained authentic fossil bones and replica bones. The parents in families who visited the spin browser (*n* = 37) were more likely to engage in science talk at the fossil exhibits than those in families who did not visit the spin browser (*n* = 46). Further, a regression analysis showed that family science talk at the fossil exhibits was predicted by parents' connections talk and children's narrative talk at the spin browser. These findings suggest that families' narratives and stories may provide an entry point for science-related talk, and encourage future study about specific links between storytelling and science understanding.

## Introduction

Around the world, stories and storytelling are part of everyday life for many children. Bruner (1986) identified narrative and storytelling as a fundamental human cognitive process, arguing that it is perhaps more natural to human thought than are logical or scientific modes of reasoning. Building on these ideas, researchers have argued that narrative may be a more effective way to communicate about science, technology, engineering, and mathematics (STEM) fields to children and students than the typical type of expository language used in textbooks (Avraamidou and Osborne, 2009; Wilson-Lopez and Gregory, 2015). Similarly, children's museum educators and early childhood educators recognize stories as a developmentally appropriate way to communicate about science and other topics with young children (Frykman, 2009). Despite growing attention to this potential connection, not much research has directly investigated the link between storytelling and science understanding.

We explored the link between stories and science in family conversations as part of an interdisciplinary collaborative project (Callanan et al., 2017) situated within a long-standing research-practice partnership (Callanan et al., 2016). Working in parallel with the design of a new children's museum exhibition, we investigated the effectiveness of a storytelling exhibit as a potential motivator for young children's engagement with science thinking in the domain of paleontology. The project focused on the potential impact of a narrative-based museum exhibit embedded within a larger children's museum exhibition regarding fossilized mammoth bones. In this NSF-funded research-practice partnership, paleontologists, science educators, and developmental science researchers worked with children's museum experts to create a developmentally appropriate exhibition focused on paleontology within a children's museum.

To provide background for the study, we first consider relevant research on children's learning through family conversations, on how narratives can support scientific thinking, and on how museum practice can support informal science learning. Finally, we introduce the study.

### Cognitive Developmental Change Through Parent-Child Conversation

Parent-child conversations in everyday interactions set the stage for children's interest and understanding about science. Research on parent-child shared book-reading has uncovered ways that family conversations can contribute to children's developing vocabulary, causal understanding, and general knowledge (Reese et al., 2010). Shirefley et al. (2020) found that family book-reading conversations can also be effective in engaging children with science practices and topics within specific fields such as astronomy.

Beyond book-reading, studies of family conversations in museums have focused on ways that family conversations introduce children to science practices such as questioning (Haden et al., 2014), observing (Eberbach and Crowley, 2017), and explaining phenomena (Crowley et al., 2001; Callanan et al., 2017, 2021). Other studies have found links from parent-child conversation to children's engagement and learning experiences (Rigney and Callanan, 2011; Haden et al., 2014). For example, Benjamin et al. (2010) found that the frequency of parents' *Wh-*questions while engaged with a museum exhibit was related to children's understanding and retention of information from the exhibit. In a recent study, Callanan et al. (2020) found that the particular timing of parents' explanatory talk was important; parents' causal talk offered when children were beginning to explore a gear exhibit predicted more systematic exploration by children. Further, Booth et al. (2020) found that parents' causal talk in conversations with young children predicts children's causal stance (i.e., their preference for causal information about novel artifacts and animals) as well as their scientific literacy.

Because parent-child explanatory conversations about science have been shown to be important for children's science learning and understanding, we asked in this study whether engagement with story-telling in a children's museum exhibit predicts more focus on explanatory science talk during the same visit.

### Stories as a Basis for Learning Science

Building on Bruner's (1986) call for cognitive science to focus on a narrative mode as well as a logical-scientific mode of human understanding, researchers have asked whether using narrative may help children better understand and connect with science topics. Communicating scientific ideas through stories may better engage non-experts with science by making the ideas more meaningful and relatable (Avraamidou and Osborne, 2009).

Further, creating narrative is arguably part of doing science: scientists such as paleontologists and astronomers put together evidence and create plausible stories of what may have happened in the past. Despite the reliance on the standard scientific method in science classrooms, Judy Scotchmoor and colleagues showed, in the website (Understanding Science, 2021), that there are many complex aspects to how science really works (see Thanukos et al., 2010). This website, which serves as a tool for science teachers and students, uses narratives of how scientific discoveries were made to illustrate how scientific arguments rely on evidence and are embedded in the scientific community and the broader world.

Listening to and creating narratives about science have been argued to relate to children's interest in science-related activities (Dahlstrom, 2014; Siegel and Cid, 2021). Further, two recent attempts to teach children complex concepts (both related to evolution) through story-telling have been quite successful (Kelemen et al., 2014; Evans et al., 2016). For example, Kelemen et al. (2014) created a storybook about natural selection and found evidence that the book supported sophisticated understanding of this abstract topic in children as young as 5 years.

### Stories in Museum Exhibit Design

Museums use narratives in their written and web-based materials to engage visitors with their activities (Frykman, 2009). Little is known about how effectively specific variations in museum exhibit design may create opportunities for families to build narratives and encourage thinking about science topics. Stories and narratives about science have been argued to relate to children's interest in science-related activities (Dahlstrom, 2014). Perhaps exhibits that provide opportunities for families to build narratives could encourage families' explanatory talk throughout the museum.

A few museum studies have shed particular light on the role of stories and narrative as a framework for children's understanding. In particular, Evans et al. (2016) found that 5–14-year-old children engaged in less anthropomorphic reasoning about species change and more reasoning about natural selection after visiting a narrative-based museum exhibit about dinosaur-bird evolution compared with visiting a control exhibit on a different topic. Further, Haden et al. (2016) explored connections between how families were prompted to interact at a building exhibit (either “build” information about how to create strong buildings, or “talk” information about having open-ended conversations with children, or both) and the narratives children told later (either after reuniting with their other parent or at home later). In this study, children's narratives were an informative measure of children's understanding because they differed depending on the prompts families were given. Both for reunion narratives and later memory narratives, children from the build + talk group talked more about engineering as a way to design and redesign their buildings, and also offered more spontaneous talk (not in answer to questions) than children in other groups. In more recent work, Pagano et al. (2019) studied children's narrative reflections about their activities in a tinkering lab at a nearby “Story Hub” exhibit. They found that families who engaged in a tinkering design challenge elaborated more in their narratives than did children who engaged in open-ended tinkering.

It is clear from the review of previous research that narratives and stories may support children's science understanding. The next step is to ask about whether a story-based exhibit might facilitate children's and families' thinking about science at related exhibits.

### Current Project

This project began with an exciting opportunity and a daunting challenge. Fossilized mammoth bones were found near Children's Discovery Museum of San Jose, and the museum was given the opportunity to build an exhibit around those bones. The NSF-funded research-practice partnership project, “Lupe's Story,” resulted, with co-PIs Jennifer Martin from Children's Discovery Museum of San Jose, Judy Scotchmoor from UC Museum of Paleontology, and Maureen Callanan from University of California, Santa Cruz. The project resulted in the permanent exhibit *Mammoth Discovery!*

The initial challenge of this project involved how to present a natural history style exhibit in a children's museum that values hands-on, active engagement with materials and phenomena. Two components of the team's solution emerged: (a) a goal of encouraging families to consider the bones as *evidence* as a way to answer questions, and (b) a focus on the developmentally appropriate activity of telling *stories*. The exhibition includes a number of opportunities for families to tell stories—including stories about the life of the animal whose remains were on display as a fossil, as well as stories about how the fossils were discovered, and how they made their way to the museum.

This study focuses on one exhibit in particular—the *Spin Browser*, a hands-on animation exhibit that allows visitors to spin a dial to view the story of the mammoth whose fossilized bones are displayed nearby. Visitors can move the dial in different speeds and directions, and can watch how the mammoth went through living, dying, being fossilized, being discovered, and being transported to the museum. The main focus of this study is whether and how engagement with story-telling at the Spin Browser might relate to scientific talk at other parts of the *Mammoth Discovery!* exhibition.

We coded families' narrative engagement with the Spin Browser, and we also coded families' explanatory talk at the exhibits that contained authentic fossils, replica bones, and large-scale skeleton replicas. Our research questions are: (1) Did families who visited the spin browser engage in more science explanatory talk at the fossil and replica bone exhibits than families who did not visit the spin browser? (2) Did family narrative talk at the spin browser predict science explanatory talk in the fossil and replica bone exhibits?

## Methods

### Participants

Eighty-three families were invited to participate as they visited Children's Discovery Museum of San Jose (San Jose, CA) on weekend days. Families agreed to be videotaped while visiting the *Mammoth Discovery!* exhibition. The exhibition was new at the time, but we also checked with families and only included those who had not yet visited. Forty-one children were in a younger group ranging from 3 to 6 years (M = 64 months) and 42 were in an older group ranging from 7 to 11 years (M = 106 months). The overall average age was 85 months. Target children included 40 boys and 43 girls. Visitors to the museum were from diverse ethnic backgrounds; families who participated described their ethnicity as White (or European-American or Caucasian): 35%, Asian (or Chinese, Chinese American, Korean, Taiwanese, or Vietnamese): 21%, South Asian (or Indian, or Asian Indian): 18%, Latino (or Hispanic, or Mexican-American): 12%, and mixed heritage (e.g., Mexican-Filipino, White-Pacific Islander): 12%. Parents were asked about their years of formal schooling as a proxy for socioeconomic level. On average, parents reported completing 16 years of school (SD = 3.05; range = 5–24 years). Half the families visited the exhibition first, and then took part in a series of activities in a research room; the other half engaged with the research room activities first and then visited the exhibit. A subset of 37 families (19 who visited the exhibit first and 18 who visited the research room first) visited the Spin Browser exhibit, 23 with girls and 14 with boys. The mean age of this group of children was 91 months.

### Procedure

Families were approached within the museum and asked to participate in a research project about how children learn with their parents. Families who agreed to participate were asked for permission to be videotaped while visiting the *Mammoth Discovery!* exhibition. Half the families were randomly selected to visit the exhibit first, and then come to a research room to complete several tasks and questionnaires; the other half of the families visited the research room first and then explored the exhibit. While in the exhibit, one member of the parent-child dyad (usually the parent) wore a lavalier remote microphone. A stationary video camera captured most of the dyad's movement through the exhibition; even when the family was not visible on camera, their audio was captured. While in the research room, parents filled out demographic and attitude questionnaires while children engaged in two tasks: a sorting task where they were chose the “same kinds of thing” from triads including fossils and human-made items, and an evidence task in the form of a storybook about finding out who spilled some paint. Finally, the parent and child were shown a “mystery object”—which was a fossilized mammoth tooth, and asked to discuss what it might be and how one would know. These measures are not considered in the present analyses.

**Figure 1:**
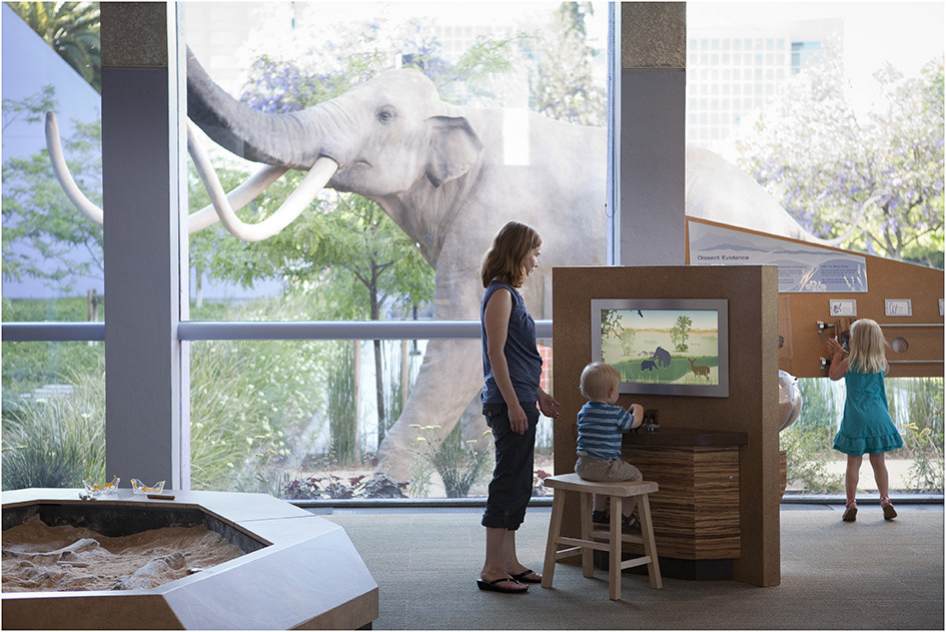
Child seated at Spin Browser exhibit in *Mammoth Discovery!* exhibition at Children's Discovery Museum of San Jose. The full-scale mammoth replica is visible outside the window and one of the Dig Pit exhibits is visible at lower left.

**Figure 2:**
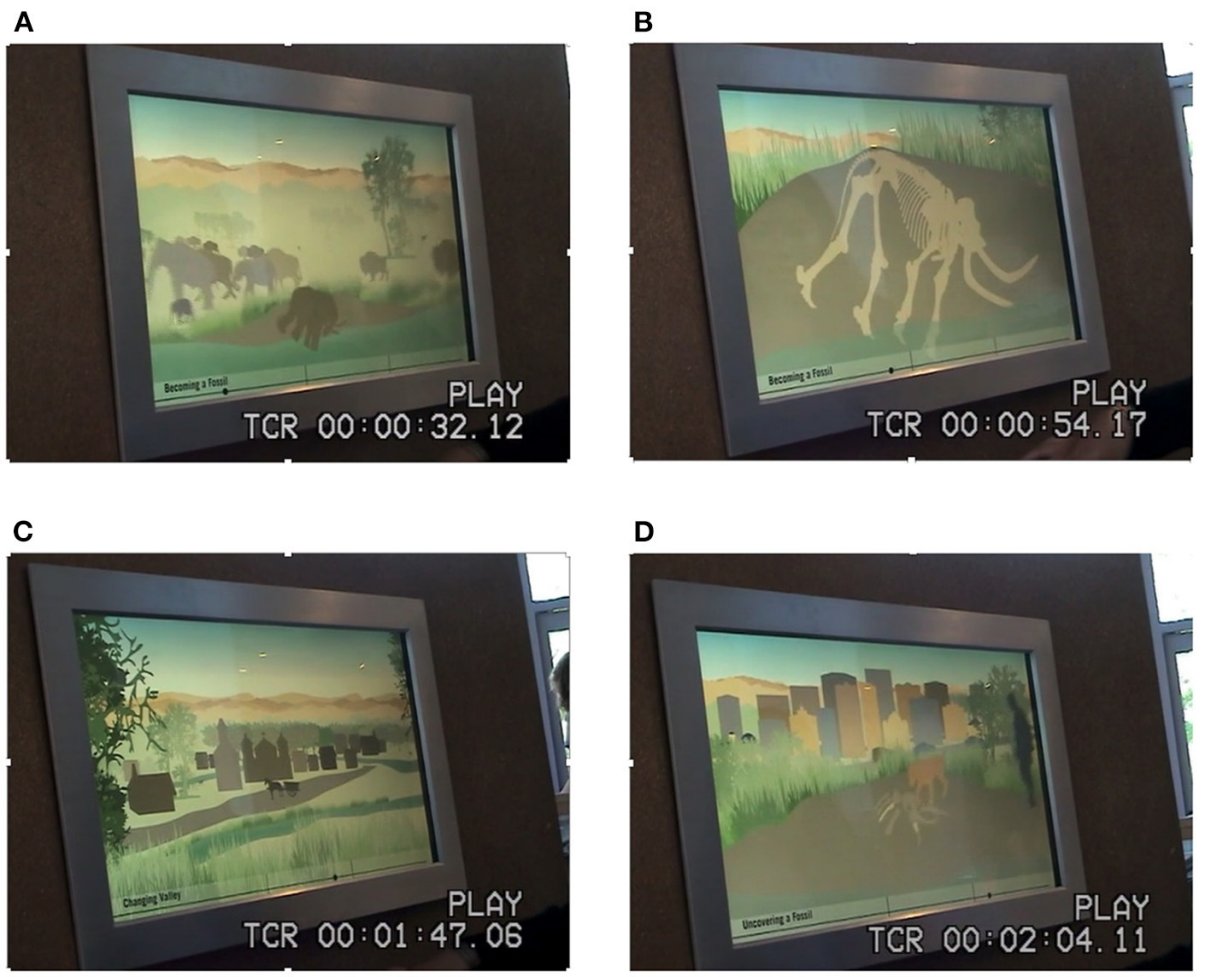
**(A–D)** Screenshots of the Spin Browser, showing **(A)** an early frame in the section on “Becoming a Fossil,” **(B)** a later frame in the section on “Becoming a Fossil,” **(C)** a frame from the section on “Changing Valley,” and **(D)** a frame in the section on “Uncovering a Fossil.”

For this study, we were particularly interested in family narrative talk occurring at the Spin Browser exhibit, an exhibit embedded within the mammoth exhibition, as shown in Figure 1. This exhibit contains a hands-on animation that allows visitors to view the story of the mammoth whose fossils were found near the museum and are displayed at the mammoth exhibit. The exhibit displays pictures that visitors can animate by turning a knob—the animation can go either forward or backward; direction and speed of turning the knob determines the direction and speed of the video. There are no signs at the exhibit but a caption is visible at the bottom left of the display and a subtle marker signals where one is in the timeline from left to right. There are three sections to the animation, each with a caption: “Becoming a Fossil,” “Changing Valley,” and “Uncovering a Fossil.” Figure 2 shows screen shot examples from each section. The exhibit design was intended to support story-telling about the mammoth's life and death, about how bones of a living animal become fossils, about changes in the local area over time, and about the discovery of the fossilized bones.

When in the *Mammoth Discovery!* exhibition, families were free to spend as much (or as little) time as they wished, and to visit any exhibits in any order. Researchers did not prompt families to visit the spin browser or any other exhibit. This meant that visits to the spin browser were not guaranteed, and that families who did visit the spin browser might do so at any time during their visit.

**Figure 3:**
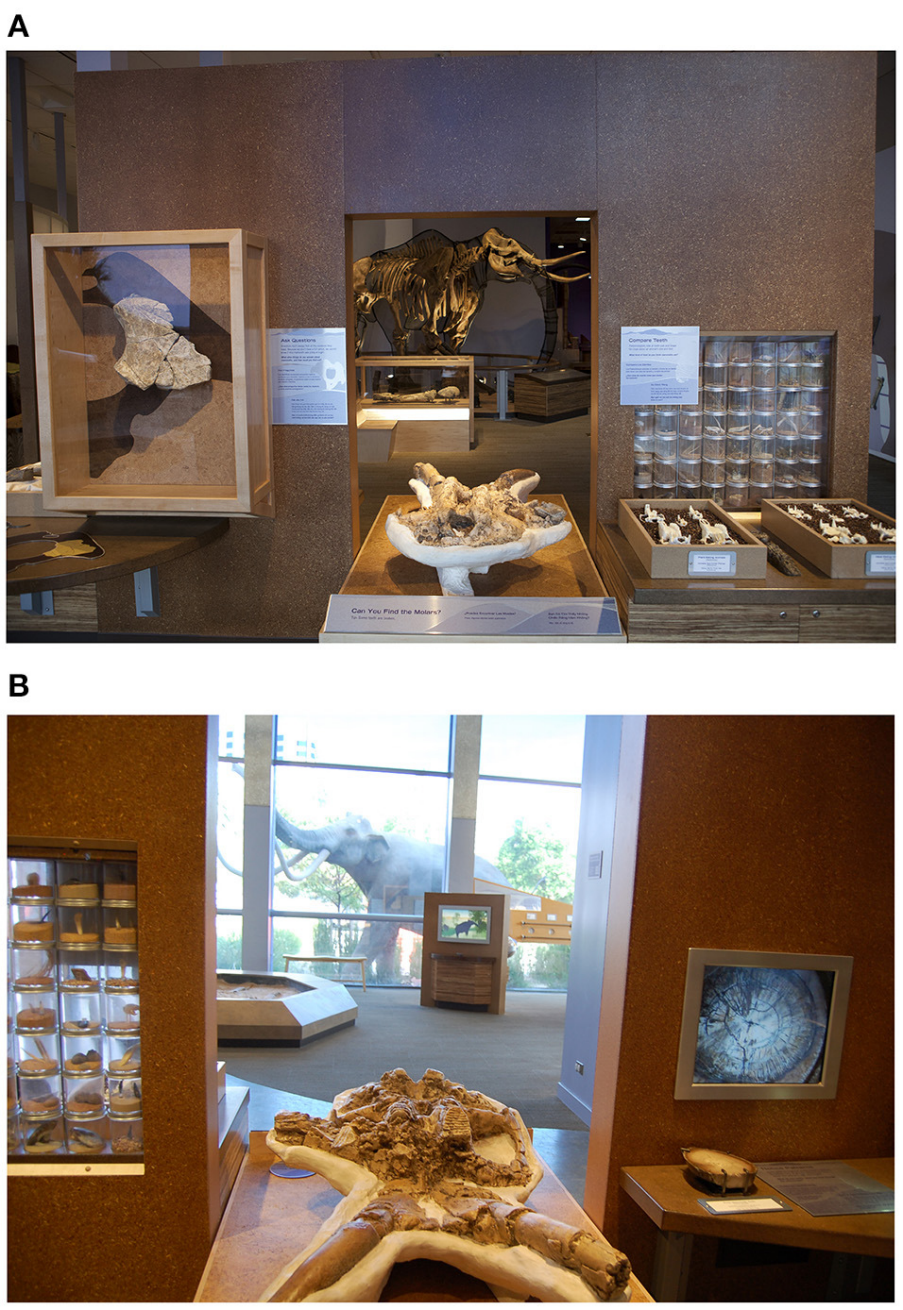
**(A)** Sample bone exhibits: Authentic pelvis bone on wall on left; replica skull in lower center; full-scale mammoth skeleton and femur bone visible through opening. **(B)** Sample bone exhibits: Replica skull lower center; Full-scale mammoth replica outside window; dig pit through opening on lower left (spin browser visible right of dig pit).

We coded families' explanatory science talk at 8 exhibits, including 3 exhibits showing authentic fossilized bones, 3 exhibits showing replicas of bones or of the full mammoth, and 2 hands-on dig pits where children could work with tools to uncover replica mammoth bones. Previous research presented some of these findings (Callanan et al., 2017). See Figure 3 for sample exhibits.

### Coding

Family visits were fully transcribed. Transcriptions captured the time that families arrived and left each exhibit as well as the verbal talk and action while visiting each exhibit. If other family members were present, their talk was transcribed as well as the target parent's and target child's. However, siblings' and other adults' talk was not coded. For the purpose of this study, we were particularly interested in whether families visited the Spin Browser exhibit, and if so, what types of talk they engaged in.

#### Narrative Talk Coding at Spin Browser Exhibit

For families who visited the Spin Browser, family interactions at the exhibit were divided into 10 second segments. Both the target parents' and children's talk were coded into four categories in terms of which category best captured each segment. Narrative talk was coded when parent or child expressed or elicited stories about the life, death, or discovery of the mammoth. Connections talk was coded when parent or child made links from the Spin Browser to other parts of the exhibition or to other aspects of children's experience. Observation was coded when parent or child observed the Spin Browser without speaking. A miscellaneous Other category captured instructions on how to use the exhibit as well as science facts about mammoths, and non-engagement with the exhibit. Using transcripts and video, two coders established inter-rater reliability on twenty percent of the videos: percent agreement was 85%, Cohen's kappa = 0.83. Table 1 provides more information about the coding categories as well as example coded utterances.

**Table 1:** Coding categories for parents' and children's talk and engagement at the spin browser exhibit (coded in 10 s segments).

**Type of talk/engagement**	**Definition**	**Examples**
Narration talk	Stories about life, death, discovery of mammoth	“*And then the bones get covered with dirt.”*
Connections talk	Links from Spin Browser to other exhibits or to previous experience	“*You wanna go see the real one?”* “*Remember in the movie Ice Age?”*
Observation	Parent or child observed without speaking	
Other	Instructional talk, off-topic talk, lack of engagement	“*Let me show you how”* “*I'm hungry”*

#### Explanatory Science Talk Coding at Fossil and Replica Exhibits

Both parents and children were coded at the utterance level in terms of the explanatory science talk they used at the eight fossil and replica exhibits. Building on previously published data (Callanan et al., 2017), our measure of explanatory science talk for these analyses included a composite total frequency of several types of explanatory talk. For our composite measure of Explanatory Science Talk we combined the frequency of *Causal explanations* about where the mammoth bones came from, what the mammoth was like when it was alive, and how scientists found the bones (e.g., “They must have dug them out of the ground!”), *Evidence talk* (e.g., “I can tell it's a mammoth because of the tusks”), *Personal connections* (e.g., “This reminds me of the elephant at the zoo”), and *Requests* for all such types of information in question form. For each of these types of talk, two coders independently coded 20% of the data and percent agreement for each type of talk ranged from 80 to 95% (Cohen's kappas ranged from 0.62 to 0.93). Table 2 provides definitions and examples for this coding scheme.

**Table 2:** Coding categories for parents' and children's explanatory science talk at the 8 fossil and replica bone exhibits (coded in number of utterances).

**Type of talk**	**Definition**	**Examples**
Causal explanation statements and requests	Explaining where mammoth bones came from, how mammoth lived, or how scientists found the bones Requesting explanations about the bones	“*They use brushes to uncover the bones.”* “*With teeth like that it must have chewed its food a lot!”* “*What do you think it looked like when it was alive?”*
Evidence statements and requests	Explicitly stating how they used evidence to draw a conclusion Requesting evidence for a claim	“*I can tell it's a mammoth because of the tusks”* “*How do they know if it's a boy or a girl?”*
Personal connections statements and requests	Making connections to previous experience or knowledge Requesting connections to previous experience or knowledge	“*This is what Uncle Ted does.” (while digging)* “*They found this near where Daddy works.”* “*What does this remind you of?”*

## Results

To investigate whether the storytelling activity at the Spin Browser encouraged scientific engagement at the fossil exhibits, we first compared the frequency of Explanatory Science Talk for families who visited the Spin Browser, compared to those who did not. Next, we investigated the patterns of types of talk at the Spin Browser, asking whether families' narrative and/or connections talk predicted their science explanatory talk at the fossil exhibits in other parts of the *Mammoth Discovery!* exhibition.

### Spin Browser Visit—Narrative Talk

We first explored the talk that families engaged in at the Spin Browser exhibit. Table 3 shows the mean number of 10-s segments coded for parents and children as narrating, connecting and observing. On average, families spent 2.7 min at the spin browser (SD = 1.39 min), with a range from 10 s to 7 min, 5 s. Overall, 92% of parents and 81% of children engaged in some narrative talk. Regarding connecting talk, 51% of parents and 24% of children made at least one connection. Preliminary analyses showed no significant differences in narrative talk or connections talk by children's gender.

**Table 3:** Parents' and children's talk and action at spin browser—Mean Number (SD) of 10-s segments coded (*n* = 37; 23 families with girls, 14 families with boys).

**Type of talk/action**	**Mean (SD)**
Children's narrative talk	4.24 (4.13)
Children's connections talk	0.35 (0.72)
Children observing	9.27 (5.99)
Parents' narrative talk	7.35 (6.64)
Parents' connections talk	1.22 (1.54)
Parents observing	3.81 (4.11)

To provide a sense of the type of narrative talk that sometimes occurred, we present an example conversation where a father, a 6-year-old child, and an older 9-year-old sibling spoke while the younger child turned the Spin Browser knob:

**Table:** 

9 year old:	There's a mammoth. Go to that side.
R6 year old:	Oh my gosh.
Dad:	There's a mammoth. Whoa. You're in the city. There's gonna be no mammoths there. Oh, that's where we're at right now.
9 year old:	That's where they dig the body.
Dad:	Mammoth, they found it near. It said on the sign they found it near, um, Guada,- some lake or river. Probably was here before.
9 year old:	Ok, so look it.
Dad:	They're showing water buffalo.
9 year old:	It's cool.
Dad:	It is, huh? Oh there's people there. Now, see it's developing. See? You're just moving down the timeline, see? Now look it, like missions…this is a small town…now it's becoming farm, farmland, the town is growing….and now a city's coming up and all of a sudden, all of a sudden, this guy comes across and finds mammoth bones here in San Jose [laughing]. What a find, huh? To be out walking with your dog and to be like “Oh look it, this looks interesting.” Call up the right people have them come out and got a, got a mammoth.

### Spin Browser Visit—Links to Explanatory Science Talk

We next asked whether families who visited the Spin Browser engaged in more Explanatory Science Talk at the fossil and replica bone exhibits compared to families who did not visit the spin browser. Table 4 shows the mean number of explanatory utterances for both parents and children, as well as the percentage of parents and children who engaged in any science talk. Comparing the explanatory science talk utterances for parents who visited vs. did not visit the Spin Browser, a *t*-test was not significant, *t*_(81)_ = 1.46, *p* = 0.15. Next we conducted a chi-square test of independence, asking whether the number of parents who used any Explanatory Science talk varied depending on whether or not they visited the Spin Browser exhibit. We found a significant relation between the two variables; parents who visited the Spin Browser were more likely to engage in Explanatory Science talk than parents who did not visit the Spin Browser, χ${}_{\left(1,\;\text{N}=83\right)}^{2}$ = 5.64, *p* = 0.018. Overall, 81% of the parents who visited the Spin Browser used some explanatory talk at the fossil exhibits, while only 56% of parents who did not visit the Spin Browser engaged in any explanatory talk at the fossil exhibits. In contrast, children's explanatory talk at the fossil exhibits did not differ depending on whether they visited the Spin Browser, χ${}_{\left(1,\;\text{N}=83\right)}^{2}$ = 1.95, *p* = 0.162.

**Table 4:** Mean number of explanatory science talk utterances by parents and children and percent of families with any science talk as a function of whether they visited the Spin Browser exhibit.

	**Mean frequency of science talk utterances**			**Percent of families with any science talk**		
**Visited Spin Browser (number of families)**	**Parents**	**Children**	**Combined**	**Parents (%)**	**Children (%)**	**Combined (%)**
Did not visit (46)	2.26	0.53	2.78	56	26	63
Did visit (37)	3.38	0.81	4.19	81	40	83
1st half of visit (22)	3.09	1.05	4.14	63	41	73
2nd half of visit (15)	3.80	0.47	4.27	100	40	100

We also asked whether parents' and children's science talk differed depending on whether they visited the Spin Browser in the first half of their exhibit visit vs. in the second half (see Table 4 for the relevant means). There were no significant differences for parents' or children's mean frequency of science talk, nor for the proportion of parents or children who engaged in science talk.

### Predicting Explanatory Talk From Spin Browser Narrative Talk

We next addressed our main question regarding whether specific types of family talk at the Spin Browser predicted explanatory talk at the fossil and replica bone exhibits. Multiple regression analyses were conducted to determine whether different types of parent and child talk at the spin browser predicted explanatory science talk at the fossil and replica bone exhibits. Because roughly half the families engaged with the researchers in the research room prior to visiting the exhibit, and the other half visited the exhibit first, we conducted preliminary regressions including order as a variable; order was not significant in these regressions and we removed it from further analyses.

In the first regression model, the outcome measure was children's explanatory science talk at the fossil exhibits. The predictors were child age and parents' years of schooling in the first block, and then adding the number of time segments coded as parents' narrative talk, children's narrative talk, parents' connection talk, children's connection talk, and parent-child observing. The regression model was marginally significant, *R*^2^ = 0.35, *F*_(7, 36)_ = 2.25, *p* = 0.058; Table 5 shows the results. Age was not a significant predictor, nor was parents' years of schooling. However, children's connection talk was significant and explained 16% percent of the variance in predicting children's explanatory science talk at the fossil exhibits (β = 0.54, *p* = 0.006), and parents' narrative talk at the Spin Browser significantly predicted children's explanatory talk at the fossil exhibits, accounting for 6.2% of the variance (β = 0.49, *p* = 0.046).

**Table 5:** Hierarchical regression: predictors of children's explanatory science talk at the fossil and replica bone exhibits.

	**B**	**BSE**	**β**
(Constant)	0.24	0.78	
Age in months	0.01	0.01	0.11
Parents' years of school	−0.04	0.07	−0.08
(Constant)	0.26	1.5	
Age in months	0.01	0.01	0.15
Parents' years of school	−0.06	0.07	−0.14
Children's narrative talk	−0.11	0.07	−0.35
Children's connections talk	0.96	0.32	0.54^*^
Parents' narrative talk	0.09	0.04	0.49^*^
Parents' connections talk	0.17	0.13	0.20
Parent and child observing	0.002	0.03	0.01

* *p <0.05*.

In a second regression model, the outcome measure was parent-child explanatory science talk, combining both parents' and children's explanatory utterances at the fossil exhibits. The same predictors were entered: children's age, parents' years of schooling, number of time segments coded as parents' narrative talk, children's narrative talk, parents' connection talk, children's connection talk, and parent-child observing. This regression model was marginally significant, *R*^2^ = 0.33, *F*_(7, 36)_ = 2.05, *p* = 0.082. In this model, shown in Table 6, the significant predictors were parents' narrative talk, β = 0.62, *p* = 0.015, and children's connection talk, β = 0.39, *p* = 0.043. Parents' narrative talk predicted 14% and children's connections talk predicted 6.3% of the variance in parent-child explanatory talk at the fossil exhibits.

**Table 6:** Hierarchical regression: predictors of combined parent-and-child explanatory science talk at the fossil and replica bone exhibits.

	**B**	**BSE**	**β**
(Constant)	10.01	3.2	
Age in months	−0.03	0.04	−0.16
Parents' years of school	−0.14	0.29	−0.09
(Constant)	3.44	5.77	
Age in months	−0.01	0.03	−0.02
Parents' years of school	−0.09	0.27	−0.06
Children's narrative talk	−0.35	0.27	−0.31
Children's connections talk	2.56	1.22	0.39^*^
Parents' narrative talk	0.45	0.17	0.62^*^
Parents' connections talk	0.68	0.49	0.22
Parent and child observing	−0.07	0.11	−0.12

* *p < 0.05*.

These results support findings that have suggested that the use of narratives may relate to children's engagement and interest in science. Specifically, in our study it was parents' narrative talk and children's connections talk that seemed to relate to families' engagement in explanatory conversations in other areas within the *Mammoth Discovery!* exhibition. This study provides some evidence that narratives may relate to other forms of science related talk, raising questions for future study about specific links between story-telling and science understanding. Understanding these links is important for the design of informal and formal science environments and fostering children's engagement in science.

## Discussion

This research project reveals ways that studies of family conversations in informal learning institutions can provide valuable insights regarding children's developing science understanding. Our findings are relevant for both research and practice; evidence regarding the hypothesis that narratives or stories may help children engage with science concepts is relevant both for theories about cognitive development and policies for creating science learning opportunities. We provide a brief summary and interpretation of our findings, consider the implications of the findings for future research and then for practice, and then end with a discussion of potential future directions.

### Summary and Interpretation of Findings

In our study, parents' narrative talk and children's connecting talk predicted explanatory science conversations in other areas within the *Mammoth Discovery!* exhibition. This study provides some evidence that families' discussions of narratives and stories may provide an entry point for forms of science-related talk, raising questions for future study about specific links between story-telling and science understanding.

These intriguing findings must be hedged, however, by acknowledging that in this type of naturalistic study it is not possible to distinguish children's engagement in storytelling or science from their interest, understanding, or learning. While it would be ideal to be able to make these distinctions, it is difficult to do so within the real life complexity of families' interactions. Indeed, we would argue that there is no perfect independent assessment of children's science understanding, and that spontaneous engagement in meaningful talk about science topics needs to be taken as seriously as test-like assessments which come with a different set of limitations. That said, we fully appreciate the need for the field to integrate these data with data from more carefully controlled studies.

It is also important to acknowledge that, because this is a naturalistic and quasi-experimental study, it is not possible to draw causal inferences from the findings. Families were not randomly assigned to visit or not visit the spin browser; instead they chose their own path and timing through the *Mammoth Discovery!* exhibition. Although it is tempting to suppose that engaging with storytelling at the spin browser exhibit might have subsequently increased families' engagement with science explanations at other exhibits, our data do not allow us to make that conclusion. Indeed, our exploration of the rough timing of spin browser visits (comparing those in the first half vs. second half of the full visit) did not yield significant differences. Hence, it is just as likely that our findings could indicate that some other factors may account for the link between storytelling and science talk. Perhaps families who engage in more storytelling talk also happen to engage in more explanatory science talk. Nevertheless, we see this observational study as an important first step; future studies should more directly address the possibility that stories may support children's science understanding.

### Links to Cognitive Developmental Science Research

These findings are consistent with previous evidence suggesting that storytelling may support children's conceptual understanding (Ganea et al., 2014; Kelemen et al., 2014). Children whose parents engaged in storytelling in their Spin Browser interactions were also likely to engage in science talk with their parents at the fossil exhibits.

The finding that children's connecting talk at the Spin Browser also predicted both children's and families' science talk at the fossil exhibits is intriguing. While not technically narrative talk, connection talk may support children in making personal meaning of the scientific objects in the exhibition. Miller et al. (1997) discuss children's personal storytelling as an important part of socialization. In similar ways, when parents bridge children's understanding by discussing personal connections to the topic under exploration, there is evidence that this can support children in making meaning of science topics (Haden et al., 2016; Callanan et al., 2017).

It is perhaps surprising that we found no gender differences in children's or parents' engagement with narrative talk. It is notable, however, that while approximately half (52%) of the participating target children were girls, 62% of the families who chose to visit the spin browser had daughters. Perhaps this storytelling exhibit was more interesting to girls, or seen as more relevant to girls by parents. This would be consistent with research suggesting that girls may have a more episodic or narrative memory style (Bemis et al., 2011). Future research should consider potential gender differences in the links between storytelling and science.

### Links to Informal Science Practice

Narrative and storytelling are argued to be natural ways to understand the world (Bruner, 1986), and developmentally appropriate ways for children to learn language, factual content, and causal connections (Melzi et al., 2011; Kelemen et al., 2014). For these reasons, storytelling is a popular technique used effectively in the design of informal learning settings and science learning materials (e.g., Evans et al., 2016; Haden et al., 2016), as well as in facilitation in museums (Pagano et al., 2020). Understanding links between storytelling and science learning is important for the design of informal and formal science environments and for fostering children's engagement in science.

The Spin Browser exhibit embedded non-verbal narrative into a hands-on exhibit, and provided opportunities for families to tell stories about the life and death of a mammoth in ways that could connect the pieces of the exhibit into a coherent whole. Our findings provide some support for the idea that such storytelling opportunities may enrich children's and parents' engagement with the science content of museum exhibits. Exploring diverse ways of connecting stories with science activities will provide valuable information about practical implications of these findings.

### Future Directions and Implications

Recent exploration of links between storytelling and science has yielded promising results. For example, several recent projects have combined storytelling or storybook reading with hands-on STEM activities, and found evidence of families' rich engagement with STEM content (Pattison et al., 2017; Tzou et al., 2019; Callanan et al., 2021). Because storytelling is an everyday cultural practice for families in many communities around the world, combining storytelling with science opens up possibilities in terms of STEM equity and inclusion (Miller et al., 2005; Solis, 2017). Our findings suggest promising directions for future work that considers family storytelling and narrative as an engaging way for children to explore and learn about science.

## Data Availability Statement

The raw data supporting the conclusions of this article can be made available by the authors.

## Ethics Statement

The study was reviewed and approved by the University of California, Santa Cruz Institutional Review Board. Written informed consent to participate in this study was provided by the participants and/or the participants' legal guardian, and verbal assent was obtained by minors who participated. Written informed consent was obtained from the individuals and minors' legal guardians for the publication of any potentially identifiable images included in this article.

## Author Contributions

JM was PI of the project. MC and JS were co-PIs. SD was head exhibit designer. MC and ML conceptualized the research. ML led data collection efforts. MD contributed to data collection and coding and wrote an undergraduate senior thesis based on some of the data. CC led coding efforts and data analysis. GS and SM contributed to data analysis. MC supervised the research and wrote the initial draft of the paper. All authors contributed to revisions.

## Conflict of Interest

The authors declare that the research was conducted in the absence of any commercial or financial relationships that could be construed as a potential conflict of interest.

## Publisher's Note

All claims expressed in this article are solely those of the authors and do not necessarily represent those of their affiliated organizations, or those of the publisher, the editors and the reviewers. Any product that may be evaluated in this article, or claim that may be made by its manufacturer, is not guaranteed or endorsed by the publisher.

## References

[B1] Avraamidou L. Osborne J. (2009). The role of narrative in communicating science. Int. J. Sci. Educ. 31, 1683–1707. 10.1080/09500690802380695

[B2] Bemis R. H. Leichtman M. D. Pillemer D. B. (2011). ‘I remember when I learned that!' Developmental and gender differences in children's memories of learning episodes. Infant Child Dev. 20, 387–399. 10.1002/icd.700

[B3] Benjamin N. Haden C. A. Wilkerson E. (2010). Enhancing building, conversation, and learning through caregiver–child interactions in a children's museum. Dev. Psychol. 46, 502–515. 10.1037/a0017822 20210509

[B4] Booth A. E. Shavlik M. Haden C. A. (2020). Parents' causal talk: links to children's causal stance and emerging scientific literacy. Dev. Psychol. 56, 2055–2064. 10.1037/dev0001108 32833470

[B5] Bruner J. S. (1986). Actual Minds, Possible Worlds. Cambridge, MA: Harvard University Press.

[B6] Callanan M. Anguiano G. Jipson J. Mahaffey E. (2021). Storybooks and science practices in family conversations about astronomy, in Paper presented at Meetings of Society for Research in Child Development, Virtual.

[B7] Callanan M. Martin J. Luce M. (2016). Two decades of families learning in a children's museum: a partnership of research and exhibit development, in Cognitive Development in Museum Settings: Relating Research and Practice, eds Sobel D. Jipson J. (New York, NY: Psychology Press), 15–35.

[B8] Callanan M. A. Castañeda C. L. Luce M. R. Martin J. L. (2017). Family science talk in museums: predicting children's engagement from variations in talk and activity. Child Dev. 88, 1492–1504. 10.1111/cdev.12886 28657198

[B9] Callanan M. A. Legare C. H. Sobel D. M. Jaeger G. J. Letourneau S. McHugh S. R.. (2020). Exploration, explanation, and parent–child interaction in museums. Monogr. Soc. Res. Child Dev. 85, 7–137. 10.1111/mono.12412 32175600 PMC10676013

[B10] Crowley K. Callanan M. A. Tenenbaum H. R. Allen E. (2001). Parents explain more often to boys than to girls during shared scientific thinking. Psychol. Sci. 12, 258–261. 10.1111/1467-9280.00347 11437311

[B11] Dahlstrom M. F. (2014). Using narratives and storytelling to communicate science with non-expert audiences. PNAS 111, 13614–13620. 10.1073/pnas.1320645111 25225368 PMC4183170

[B12] Eberbach C. Crowley K. (2017). From seeing to observing: how parents and children learn to see science in a botanical garden. J. Learn. Sci. 26, 608–642. 10.1080/10508406.2017.1308867

[B13] Evans E. M. Weiss M. Lane J. L. Palmquist S. (2016). The spiral model: integrating research and exhibit development to foster conceptual change, in Cognitive Development in Museum Settings: Relating Research and Practice, eds Sobel D. M. Jipson J. L. (New York, NY: Psychology Press), 36–64.

[B14] Frykman S. (2009). Stories to tell? Narrative tools in museum education texts. Educ. Res. 51, 299–319. 10.1080/00131880903156898

[B15] Ganea P. A. Canfield C. F. Simons-Ghafari K. Chou T. (2014). Do cavies talk? The effect of anthropomorphic picture books on children's knowledge about animals. Front. Psychol. 5:283. 10.3389/fpsyg.2014.00283 24782793 PMC3989584

[B16] Haden C. A. Cohen T. Uttal D. Marcus M. (2016). Building learning: narrating and transferring experiences in a children's museum, in Cognitive Development in Museum Settings: Relating Research and Practice, eds Sobel D. Jipson J. (New York, NY: Psychology Press), 84–103.

[B17] Haden C. A. Jant E. A. Hoffman P. C. Marcus M. Geddes J. R. Gaskins S. (2014). Supporting family conversations and children's STEM learning in a children's museum. Early Child. Res. Q. 29, 333–344. 10.1016/j.ecresq.2014.04.004

[B18] Kelemen D. Emmons N. A. Seston Schillaci R. Ganea P. A. (2014). Young children can be taught basic natural selection using a picture-storybook intervention. Psychol. Sci. 25, 893–902. 10.1177/0956797613516009 24503874

[B19] Melzi G. Schick A. R. Kennedy J. L. (2011). Narrative elaboration and participation: two dimensions of maternal elicitation style. Child Dev. 82, 1282–1296. 10.1111/j.1467-8624.2011.01600.x 21557744

[B20] Miller P. J. Cho G. E. Bracey J. R. (2005). Working-class children's experience through the prism of personal storytelling. Hum. Dev. 48, 115–135. 10.1159/000085515

[B21] Miller P. J. Wiley A. R. Fung H. Liang C. H. (1997). Personal storytelling as a medium of socialization in Chinese and American families. Child Dev. 68, 557–568. 10.2307/1131678 9249966

[B22] Pagano L. C. Haden C. A. Uttal D. H. (2020). Museum program design supports parent-child engineering talk during tinkering and reminiscing. J. Exp. Child Psychol. 10.1016/j.jecp.2020.104944. [Epub ahead of print]. 32791381

[B23] Pagano L. C. Haden C. A. Uttal D. H. Cohen T. (2019). Conversational reflections about tinkering experiences in a children's museum. Sci. Educ. 103, 1493–1512. 10.1002/sce.21536

[B24] Pattison S. Svarovsky G. Gontan I. Corrie P. G. Benne M. Weiss S.. (2017). Head start on engineering: teachers, informal STEM educators, and learning researchers collaborating to engage low-income families with engineering. Connect. Sci. Learn. 1. Available online at: https://www.nsta.org/connected-science-learning/connected-science-learning-october-december-2017-0/head-start

[B25] Reese E. Sparks A. Leyva D. (2010). A review of parent interventions for preschool children's language and emergent literacy. J. Early Childhood Literacy 10, 97–117. 10.1177/146879840935698734208785

[B26] Rigney J. C. Callanan M. A. (2011). Patterns in parent–child conversations about animals at a marine science center. Cogn. Dev. 26, 155–171. 10.1016/j.cogdev.2010.12.002

[B27] Shirefley T. A. Castañeda C. L. Rodriguez-Gutiérrez J. Callanan M. A. Jipson J. (2020). Science conversations during family book reading with girls and boys in two cultural communities. J. Cogn. Dev. 21, 551–572. 10.1080/15248372.2020.1797750

[B28] Siegel D. Cid C. (2021). Rural Family STEM Learning Through Story Writing. Paper presented at meetings of Society for Research in Child Development.

[B29] Solis G. (2017). Mexican and European heritage families' elaborations during two storytelling activities (Publication No. 10688313) (Doctoral dissertation). University of California, Santa Cruz, CA, United States. ProQuest Dissertations & Theses Global.

[B30] Thanukos A. Scotchmoor J. Caldwell R. Lindberg D. (2010). Science 101: building the foundations for real understanding. Science 330, 1764–1765. 10.1126/science.1186994 21127217

[B31] Tzou C. Meixi Suárez E. Bell P. LaBonte D. Starks E. Bang M. (2019). Storywork in STEM-art: making, materiality and robotics within everyday acts of indigenous presence and resurgence. Cogn. Instr. 37, 306–326. 10.1080/07370008.2019.1624547

[B32] Understanding Science (2021). University of California Museum of Paleontology. Available online at: http://www.understandingscience.org (accessed January 3, 2021).

[B33] Wilson-Lopez A. Gregory S. (2015). Integrating literacy and engineering instruction for young learners. Read. Teach. 69, 25–33. 10.1002/trtr.1351

